# MULoc-target: Targeting peptide classification and detection using a protein language model

**DOI:** 10.1093/bib/bbaf436

**Published:** 2025-08-27

**Authors:** Yuexu Jiang, Duolin Wang, Shuai Zeng, Yichuan Zhang, Lei Jiang, Mahdi Pourmirzaei, Negin Manshour, Farzaneh Esmaili, Weinan Zhang, Ian M Møller, Dong Xu

**Affiliations:** Department of Chemical and Materials Engineering, University of Kentucky, 177, 512 Administration Dr F. Paul Anderson Tower, Lexington, KY 40506, United States; Department of Electrical Engineering and Computer Science and Christopher S. Bond Life Sciences Center, University of Missouri, 1201 Rollins St, Columbia, MO 65211, United States; Department of Electrical Engineering and Computer Science and Christopher S. Bond Life Sciences Center, University of Missouri, 1201 Rollins St, Columbia, MO 65211, United States; Department of Electrical Engineering and Computer Science and Christopher S. Bond Life Sciences Center, University of Missouri, 1201 Rollins St, Columbia, MO 65211, United States; Department of Electrical Engineering and Computer Science and Christopher S. Bond Life Sciences Center, University of Missouri, 1201 Rollins St, Columbia, MO 65211, United States; Department of Electrical Engineering and Computer Science and Christopher S. Bond Life Sciences Center, University of Missouri, 1201 Rollins St, Columbia, MO 65211, United States; Department of Electrical Engineering and Computer Science and Christopher S. Bond Life Sciences Center, University of Missouri, 1201 Rollins St, Columbia, MO 65211, United States; Department of Electrical Engineering and Computer Science and Christopher S. Bond Life Sciences Center, University of Missouri, 1201 Rollins St, Columbia, MO 65211, United States; Department of Electrical Engineering and Computer Science and Christopher S. Bond Life Sciences Center, University of Missouri, 1201 Rollins St, Columbia, MO 65211, United States; Department of Electrical Engineering and Computer Science and Christopher S. Bond Life Sciences Center, University of Missouri, 1201 Rollins St, Columbia, MO 65211, United States; Department of Molecular Biology and Genetics, Aarhus University, Forsøgsvej 1, Slagelse DK-4200, Denmark; Department of Electrical Engineering and Computer Science and Christopher S. Bond Life Sciences Center, University of Missouri, 1201 Rollins St, Columbia, MO 65211, United States

**Keywords:** protein localization, protein targeting peptide, protein language model, deep learning, parameter efficient fine tuning, peptide classification and detection

## Abstract

Protein targeting, often guided by targeting peptides, is a critical biological process that directs proteins to their specific cellular destinations, ensuring proper cellular functionality and organization. Accurate classification and detection of targeting peptides are fundamental to understanding protein sorting mechanisms. This study introduces MULoc-Target, a novel deep-learning method designed to detect and classify targeting peptides in eukaryotic proteins. To support its development and evaluation, we curated a benchmark dataset comprising eight types of eukaryotic targeting peptides with manually curated annotations. Comprehensive evaluations on this dataset and external datasets from the literature demonstrate that MULoc-Target achieves state-of-the-art or competitive performance in detecting and classifying targeting peptides. Additionally, it enables the extraction of enriched motif patterns, offering valuable insights into their properties and the underlying targeting mechanisms. The identified motifs align closely with established biological features, further validating MULoc-Target's capabilities. A web server for MULoc-Target is integrated into our MULocDeep localization suite as a new toolkit, publicly accessible at https://mu-loc.org/MULoc-Target, and the inference code is available at https://github.com/yuexujiang/MULoc-Target.

## Introduction

Protein targeting, also known as protein sorting, is a fundamental biological process that directs newly synthesized proteins to their specific destinations within or outside the cell [1]. Accurate protein sorting is essential for maintaining cellular homeostasis, and defects in this process have been implicated in various diseases [2, 3]. Proteins typically possess short amino acid sequences, often located at one terminus, which function analogously to postal codes, directing them to their proper localization within the cell [4]. Understanding the mechanisms underlying protein sorting provides valuable insights into cellular biology and offers potential therapeutic targets for correcting sorting-related dysfunctions [5, 6].

Following transcription, protein translation bifurcates into two primary pathways based on the protein’s ultimate destination. Non-secreted proteins are synthesized by free ribosomes in the cytosol [7]. Without targeting peptides, these proteins remain in the cytosol and attain their mature functional forms. Conversely, proteins containing specific targeting peptides are directed to various intracellular compartments. These targeting peptides serve as molecular signals that facilitate the translocation of proteins to their designated locations. Key targeting signals include mitochondrial transit peptides (MT), chloroplast transit peptides (CH), thylakoidal transit peptides (TH), peroxisomal targeting signals (PTS), nuclear localization signals (NLS), and nuclear export signals (NES). The signals exhibit distinct localization patterns within protein sequences: MT, CH, and TH are typically located at the N-terminus; PTS can be found at either terminus; NLS and NES may consist of multiple discontinuous segments and can be present at various positions within a protein sequence.

Secreted proteins are translated by ribosomes associated with the endoplasmic reticulum (ER) [7]. Most proteins destined for the secretory pathway possess an N-terminal signal peptide (SP) that directs their translocation into the ER lumen upon synthesis. These proteins are transported from the ER to the Golgi apparatus for further processing and sorting. Proteins containing a specific four-amino-acid retention signal at their C-terminus are retained within the ER [8]. Without such a retention signal, proteins are trafficked to various destinations, including extracellular secretion, incorporation into the plasma membrane, or localization to the lumen or membrane of the Golgi apparatus and endosomes.

Several deep-learning methods have been developed to predict and detect targeting peptides. Despite significant advancements, challenges remain in targeting peptide detection. Specialized predictors, such as TargetP 2.0 [9], can detect four types of N-terminal targeting peptides, while SignalP [10] focuses on the detailed classification of different signal peptides. However, these methods cover only approximately half of the common targeting peptide types discussed above. Additionally, some general protein subcellular localization prediction methods can be adapted for targeting peptide detection through multitasking strategies or by leveraging attention mechanisms. Representative methods in this category include DeepLoc 2 [11] and MULocDeep [12, 13]. The DeepLoc method utilizes a protein language model and simultaneously addresses both localization prediction and sorting signal prediction, but signal position detection is not provided. MULocDeep predicts protein localization at both subcellular and suborganellar levels and quantifies amino acid contributions using attention scores, which can be used to infer the presence of targeting peptides. However, attention scores may not be sufficiently sensitive to provide precise position of targeting signals within protein sequences.

In this paper, we propose a novel deep learning method, MULoc-Target, designed to comprehensively classify targeting peptides and, importantly, provide their precise position within protein sequences. Unlike existing methods that only consider N-terminal signals and limit the input to the first 200 amino acids, MULoc-Target leverages the ESM2 [14] protein language model as its backbone to encode protein sequence fragments into fixed-length representations, which are subsequently assembled to reconstruct the original protein. This approach allows MULoc-Target to process proteins without sequence length constraints and to detect signals anywhere on the protein sequence. To address data imbalance and the issue of small sample sizes for underrepresented classes, we implemented a data augmentation process based on a binomial distribution with varying probabilities for signal and non-signal amino acids.

We systematically curated a dataset (UniProt-EC7) from the UniProt database [15] (Materials and Methods), comprising proteins from eukaryotic species with annotated targeting peptide types and positions. Evaluations using this dataset, along with external datasets from other studies, demonstrate that MULoc-Target outperforms major existing methods in both targeting peptide type prediction and position detection. The datasets, source code, and web server for MULoc-Target are all publicly available, facilitating its adoption and further development by the research community.

## Materials and methods

### UniProt-EC7 dataset creation

We collected protein sequence data and localization annotations for animals, plants, and fungi from UniProt [15] database release 2023_11. We downloaded all protein sequences with existence codes indicating evidence at the protein level and then excluded proteins originally encoded in mitochondria, plastids, or chloroplasts. Proteins with transit peptide, signal peptide, and motif annotations with reference and manually curated evidence codes (ECO:0000269 for published experimental evidence, ECO:0000303 for non-traceable author statement, ECO:0000305 for curator inference evidence, ECO:0000250 sequence similarity evidence, ECO:0000255 for sequence model evidence, ECO:0000312 for imported information evidence, ECO:0007744 for combinatorial evidence) were selected, including 8 targeting peptide classes. The sample numbers in each targeting peptide class are listed in Table S1. More precisely, the sample numbers of different evidence codes are summarized in Table S2. We used ggsearch36 [16] (v36.3.8) to calculate pairwise similarity scores for assessing sequence identity within each group. Each group was further partitioned into five subsets to avoid data leakage, grouping similar sequences within each subset while ensuring ˂30% sequence similarity between proteins from different subsets. These five subsets in each group were assigned partition numbers for generating training, validation, and test datasets through 5-fold cross-validation. The dataset can be accessed from our GitHub repository.

### Data augmentation

The data augmentation process in this implementation is designed to generate diverse and biologically plausible variations of protein sequences while maintaining their essential structural and functional characteristics. This is achieved through a targeted random mutation approach and systematic augmentation of training samples. The process is as follows:

Random Mutations Based on Binomial Distribution: Each amino acid in a sequence is assigned a probability of mutation, determined by whether it is part of a "positive" or "negative" region in the sequence. Positive regions, which correspond to annotated targeting peptide areas, are mutated with a mutation rate set as 0.1, while non-targeting regions are mutated with a mutation rate set as 0.3. Using a binomial distribution, specific positions are selected for mutation, and the amino acids at these positions are replaced with randomly chosen amino acids from a predefined standard amino acid set.

Iterative Augmentation: Each sequence in the dataset is augmented multiple times based on the weight of its class in the dataset. This ensures that underrepresented classes are adequately sampled, addressing potential class imbalance. The augmentation rate is proportional to the class weight, calculated using a predefined configuration.

Control of Sequence Identity: Augmented sequences are carefully split into training and validation folds with sequence identity control, ensuring no highly similar sequences across folds. This maintains the robustness and generalizability of the model during training and evaluation.

Integration into the Dataset: Augmented sequences, along with their corresponding annotations, are added to the dataset. In each epoch, the augmented data are generated from a new random process. This significantly increases the size of the training dataset while preserving key biological signals and reducing noise.

### ESM2 sequence encoder

Our sequence encoder was built upon the pretrained ESM2 model [14]. Due to limitations in computational resources and model capacity, we chose the ESM2-t33_650M_UR50D variant as our foundational pre-trained language model (PLM), which consists of 650 million parameters. Specifically, we began by tokenizing the input protein fragments using one-hot encoding for each amino acid and then processed them through 33 layers of Transformer encoders. Each positional embedding had a dimension of 1280. In this process, we added a BEGIN token (‘<cls>’) and an END token (‘<eos>’) to the sequence, which were input into the Transformer alongside the amino acid tokens. Additionally, a ‘<pad>’ token was used to pad sequences shorter than the predefined fragment length of 1022 amino acids. The Transformer layers produced an output tensor with a 1280-dimensional vector for each residue, excluding the embeddings for the BEGIN and END tokens as well as any padding. To reconstruct the fragment representations into a complete protein sequence, we introduced an overlap of 200 amino acids between consecutive fragments and calculated the mean of the embeddings for the overlapping amino acids to merge them seamlessly.

### MULoc-target training procedure

Training MULoc-target involved optimizing the predicted signal strength matrix against ground truth signals using cross-entropy losses. We designed two types of loss functions. One was a vertical residue-level loss function. Since one residue can only be used in at most one type of targeting peptide, we used weighted categorical cross-entropy to calculate the residue-level loss. The weight is measured based on the frequency of being selected as the maximum value among all targeting peptides at each amino acid position. For positions that do not have a targeting signal (or signals not covered by our model), we set a “other class” at the residue level, which is calculated as $1-\max \left( residue\ level\ vector\right)$. Additionally, we include a protein-level loss function by horizontally selecting the maximum signal value across amino acid positions and then comparing it with the ground truth targeting peptide class label using a weighted binary cross-entropy loss. Binary cross-entropy allows our model to perform multi-label prediction for targeting peptide type. The weight is measured based on the sample numbers among all the targeting peptide classes. Similarly, we set a “other class” at the protein level, which is calculated as $1-\max \left( protein\ level\ vector\right)$. This other class is for proteins for which our model did not identify any targeting peptide.

Each task head is optimized individually to ensure specialized learning for its targeting peptide type. The model is trained for up to 200 epochs with an early stopping set at 30 epochs of no improvement. If a task head does not improve within this timeframe, the backbone and the corresponding task head are frozen to prevent overfitting, and training continues for the remaining task heads until all heads are optimized or frozen.

### PEFT on the sequence encoder

**Figure 1:**
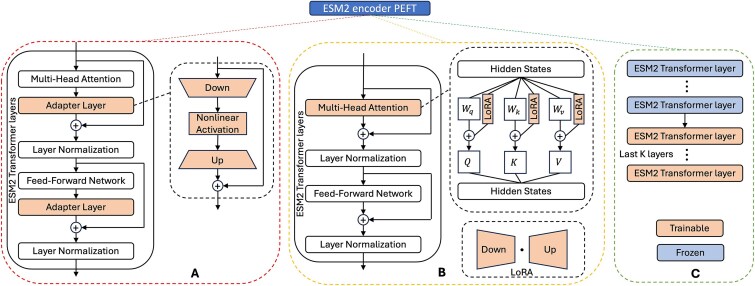
Schematic implementation of PEFT methods on the ESM2 encoder. (A) MULoc-target integrates trainable adapter layers into the transformer layers of the ESM2 model. (B) MULoc-target incorporates trainable low-rank decomposition matrices within the transformer layers of the ESM2 model. (C) MULoc-target fine-tunes only the last K transformer layers in the ESM2 model while freezing all other parameters. In this study, K was set to 2.

Our Parameter-Efficient Fine-Tuning (PEFT) strategy encompasses training methods that introduce targeted and typically parameter-efficient adjustments to an existing model. These modifications help lower the computational demands and memory usage compared to fully fine-tuning the entire model. Specifically, we applied three PEFT techniques, fine-tuning the last K layers, LoRA [17], and adapter tuning [18], to the sequence encoder, as illustrated in Fig. 1. Below are the detailed descriptions of each approach:

Fine-tuning last *K* layers: The ESM2 backbone model comprises 33 Transformer layers in total. In our approach, “fine-tuning the last *K* layers” involves updating only the final *K* = 2 Transformer layers while keeping the remaining layers frozen. Here, *K* is treated as a hyperparameter within our experimental setup.

LoRA: LoRA stands for Low-Rank Adaptation, a technique that maintains the pretrained model weights unchanged and introduces trainable low-rank decomposition matrices into each Transformer layer. This method significantly reduces the number of parameters that need to be trained for downstream tasks. Specifically, for a pretrained weight matrix ${W}_0\in{\mathbb{R}}^{d\times k}$, its update $\Delta W$ is expressed as a low-rank decomposition $\Delta W= BA,\kern0.5em \mathrm{where}\ B\in{\mathbb{R}}^{d\times r},A\in{\mathbb{R}}^{r\times k}$, with the rank $r$ being much smaller than $\min \left(d,k\right)$. During training, ${W}_0$ remains frozen, and only matrices $A$ and $B$ are trainable. For an input $x$, the LoRA-enhanced forward pass is computed as:


(1) \begin{equation*} h={W}_0+\Delta Wx={W}_0+ BAx \end{equation*}

In our implementation, these low-rank decomposition matrices were integrated into the query (Q), key (K), and value (V) within the self-attention modules of the last 16 Transformer layers of ESM2 (Fig. 1).

Adapter tuning involves adding adapter modules to the Transformer layers of the ESM2 model. Following the methodology proposed by Houlsby *et al*. [18], each adapter module is inserted twice within a single Transformer layer: once after the self-attention projection and once after the two feed-forward networks. Each adapter module features a bottleneck architecture and a skip connection. The bottleneck structure first compresses the input data into a lower-dimensional space and then reconstructs it back to the original dimension. This design allows the adapter modules to introduce only a minimal number of additional parameters relative to the original attention and feed-forward layers. In our setup, adapters were incorporated into the last five Transformer layers of ESM2.

### Evaluation criteria

We used Matthew’s correlation coefficient (MCC) and F1 score to evaluate targeting peptide classification performance. For unbalanced datasets, measurements such as accuracy (ACC), recall and precision would introduce bias and overestimate a method’s performance. MCC considers true and false positives and negatives, and is generally regarded as a balanced measure even if the classes are of very different sizes. The F1 score is a widely used metric for evaluating the performance of classification models, particularly in cases where the data are imbalanced. It is the harmonic mean of precision and recall, providing a single measure that balances these two important metrics. The calculations of MCC and F1 scores are shown in Eqs. (2) and (3):


(2) \begin{equation*} MCC=\frac{TP\ast TN- FP\ast FN}{\sqrt{\left( TP+ FP\right)\left( TP+ FN\right)\left( TN+ FP\right)\left( TN+ FN\right)}} \end{equation*}

where *TP, FP, TN, FN* are true positive, false positive, true negative and false negative predictions, respectively.


(3) \begin{equation*} F1\ score=2\ast \frac{Precision\ast Recall}{Precision+ Recall} \end{equation*}

Two criteria were used for the evaluation of targeting peptide detection in different scenarios. For targeting peptides including MT, CH, TH, SP, PLS, ER, one end is always the N-terminus or C-terminus. Therefore, we used accuracy to measure the proportion of exact correct detection of the other end among the total predictions of the corresponding targeting peptide class.

For NLS and NES where the signals form variant length segments along the protein sequence, we used Segment Overlap [19] (SOV score) to measure the overlap between ground truth signals and MULoc-Target predicted signals. The SOV measure was originally introduced in the CASP (Critical Assessment of protein Structure Prediction) experiments to evaluate how well predicted secondary structure segments match the experimentally determined ones [20]. It can be generalized as a metric designed to capture both how many residues you get right in a segment and how well you get the segment boundaries correct. It provides a segment-oriented view of accuracy, more nuanced than a simple residue-level or overlap-based metric like Jaccard [21]. The simplified core formula is


(4) \begin{equation*} SOV=\frac{\sum_{i=1}^n\min \left( OV(i),{\alpha}_i\ast union(i)\right)}{\sum_{i=1}^n\mid{S}_{obs,i}\mid } \end{equation*}

where ${\alpha}_i=1-\frac{\left\Vert{S}_{obs,i}\mid -\mid{S}_{pred,i}\right\Vert }{union(i)}$, ${S}_{obs,i}$= the $i$-th observed segment of a given signal, ${S}_{pred,i}$= the predicted segment that overlaps (at least partially) with ${S}_{obs,i}$, $OV(i)=\mid{S}_{obs,i}\cap{S}_{pred,i}\mid$, the number of overlapping residues between the observed and predicted segments $i$. $union(i)=\mid{S}_{obs,i}\cup{S}_{pred,i}\mid$, the total number of unique residues covered by either the observed or the predicted segment $i$.

### AlphaFold 3 web application

To validate the binding region between two proteins, we used the web server of AlphaFold 3 at https://alphafoldserver.com/. For each dimer complex, we upload the sequences of the two proteins. Once the job is finished, we download the results, which contain the atomic coordinates in the CIF format of the top five predicted structures. We converted the CIF files into PDB files using MMCIFParser and PDBIO python packages. Then we used PDBParser to parse the complex structure. For each pair of amino acids between the two proteins, we considered them to be interacting if the distance between any of their atoms was ˂5 Å. We output the binding amino acid indices and numbers of the two proteins, respectively.

## Results

### MULoc-target architecture

**Figure 2:**
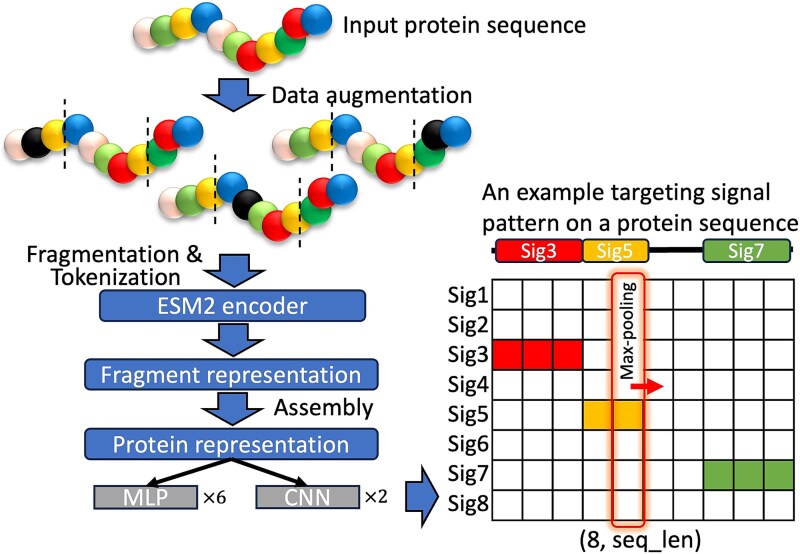
The schematic architecture of MULoc-target. The model backbone utilizes evolutionary scale modeling (ESM2), while CNN and MLP are used for task-specific classifiers. The input to MULoc-target is a protein sequence. The model processes this sequence and generates an output matrix where each row corresponds to a specific targeting peptide type, and each column represents the predicted signal strength for each amino acid position within the protein sequence. During inference, MULoc-target applies vertical max-pooling to each amino acid position, selecting the highest signal strength across all targeting peptide types. If one type of targeting peptide has the highest signal, it means the protein contains such a type of targeting peptide. This approach enables precise detection of targeting signals by identifying the most probable targeting peptide at each position in the protein sequence, and simultaneously predicts targeting peptide types.

As illustrated in Fig. 2, MULoc-Target processes protein sequences as input through a carefully designed architecture to classify and locate targeting peptides accurately. During training, MULoc-Target begins with a data augmentation step to address class imbalance (Materials and Methods). This process introduces variants to protein sequences and generates additional training samples inversely proportional to the original class distribution, thereby improving model robustness and performance, particularly for underrepresented classes. For inference, the data augmentation step is skipped.

Protein sequences are then divided into fragments of fixed length, specifically 1022 amino acids, to ensure uniform input sizes for processing. Each fragment is tokenized and fed into the ESM2 (650 M parameters) protein language model to generate fragment representations (Materials and Methods). These representations capture rich contextual relationships among amino acids, which are crucial for accurate targeting peptide predictions. The fragment representations are then reassembled to reconstruct the full protein sequence representation, preserving both local and global structural information.

The assembled protein representation is passed through eight task-specific prediction heads, each dedicated to a specific targeting peptide type. Convolutional neural networks (CNNs) are employed for predicting NLS and NES due to their ability to capture local sequence patterns of variable length. For the remaining six targeting peptide types, which are located at either the N-terminus or C-terminus of the sequence, multi-layer perceptrons (MLPs) are used to predict the location of the signal peptide's opposite end. Each task head generates a prediction vector equal in length to the original protein sequence, representing the signal strength at each amino acid position for its corresponding targeting peptide.

The outputs from the eight task heads are combined into a matrix, where each row represents a specific targeting peptide type, and each column corresponds to an amino acid position in the protein sequence. The values in the matrix indicate the predicted signal strength for each targeting peptide type at each position. We noted that one specific amino acid cannot be utilized in more than one targeting peptide. In other words, there can be no overlap between targeting peptides. Thus, MULoc-Target applies vertical max-pooling to the signal strength matrix for inference, selecting the highest value at each amino acid position across all targeting peptide types. This eliminates the need for threshold-based comparisons and ensures precise identification of the most probable targeting peptide at each position. For example, as shown in Fig. 2, distinct regions of the protein sequence may display maximum signals corresponding to different targeting peptides, such as red for Signal 3, yellow for Signal 5, and green for Signal 7. These signals collectively form the targeting peptide patterns. The details of the training process for MULoc-Target are illustrated in Materials and Methods.

### Parameter-efficient fine-tuning on ESM2 encoder

We employed several parameter-efficient fine-tuning (PEFT) methods to train the model, including fine-tuning the last *K* layers (*K = 2*) of the ESM2 model, incorporating adapter layers [18], and utilizing Low-Rank Adaptation (LoRA) techniques [17]. These PEFT methods are designed to adapt pre-trained models to specific tasks with minimal computational and storage overhead.

LoRA [17] achieves this by introducing low-rank matrices into the existing layers of the model, capturing task-specific nuances without modifying the original pre-trained weights. This approach significantly reduces the number of trainable parameters, making fine-tuning more efficient and scalable. Conversely, adapter-based methods [18] incorporate small, trainable bottleneck layers into the network architecture. These layers enable the model to learn new task-specific representations while keeping most pre-trained parameters fixed. Adapters provide a modular and flexible approach for specializing models for diverse applications without requiring extensive re-training. The schematics illustrating the application of these PEFT methods are shown in Fig. 1, with implementation details described in Materials and Methods.

We evaluated the performance of the different PEFT methods on our UniProt-EC7 dataset and present the cross-validation results in Table 1. The evaluation metrics for targeting peptide classification included the Matthews correlation coefficient (MCC) and F1 score. Two scenarios were considered for targeting peptide position detection. For peptides located at either the N-terminus or C-terminus, we measured performance using cleavage site accuracy, which quantifies the accuracy of detecting the other terminus of the peptide. For Nuclear Localization Signals (NLS) and Nuclear Export Signals (NES), we used the Segment Overlap Measure [19] (SOV score) to assess the overlap between the predicted and ground-truth signal positions (see Materials and Methods for evaluation criteria).

**Table 1:** Cross-validation performance of PEFT method on the UniProt-EC7 dataset.

Targeting peptides	Fine-tuning last *K* layers			Adapter			LoRA		
	Targeting peptide Classification		Targeting peptide detection	Targeting peptide Classification		Targeting peptide detection	Targeting peptide Classification		Targeting peptide detection
	MCC	F1	Cleavage site accuracy	MCC	F1	Cleavage site accuracy	MCC	F1	Cleavage site accuracy
ER	**0.814**	**0.807**	0.985	0.800	0.717	0.874	0.771	0.701	**0.992**
PTS	**0.604**	**0.591**	**0.885**	0.455	0.440	0.800	0.569	0.544	0.846
MT	0.936	0.944	**0.565**	**0.951**	**0.957**	0.268	0.947	0.954	0.431
SP	0.930	0.973	**0.868**	0.937	0.976	0.789	**0.941**	**0.978**	0.844
CH	**0.962**	**0.964**	**0.355**	0.958	0.961	0.186	0.932	0.934	0.303
TH	**0.798**	**0.783**	**0.936**	0.775	0.752	0.891	0.766	0.741	0.867
	MCC	F1	SOV score	MCC	F1	SOV score	MCC	F1	SOV score
NLS	0.776	0.798	0.658	0.651	0.668	0.624	**0.781**	**0.789**	**0.661**
NES	0.385	0.354	0.277	0.283	0.289	0.221	**0.409**	**0.464**	**0.367**
Average	**0.776**	**0.777**	**0.691**	0.726	0.720	0.582	0.765	0.763	0.664
Weighted average	0.887	0.915	**0.749**	0.869	0.893	0.640	**0.893**	0.915	0.715

Best results for each task are shown in bold.

Note: Averaging the cleavage site accuracy and SOV score is only meaningful for comparing values across different configurations; it has no statistical significance. The weighted average is based on the sample number of each class in the dataset.

As shown in Table 1, fine-tuning the last *K* layers demonstrated overall superior performance compared to other PEFT methods. Consequently, all subsequent results in this study are based on this fine-tuning strategy. Using this model configuration, we further evaluated MULoc-Target on a subset of the UniProt-EC7 dataset where all samples in this subset have experimental verification evidence code ECO:0000269. The results are shown in Table S3. The purpose of making such an evaluation is to remove the noise from some low-quality samples without experimental verification. However, this would leave too few samples for some classes to give a statistically robust evaluation. We also did an ablation test to justify the contribution of data augmentation, the weight on the residue-level loss function, and the weight on the protein-level loss function (see Materials and Methods for the details of the weight definition). The results are presented in Table S4, which shows that using all three strategies achieved the best overall performance. The models without class weight or data augmentation achieved comparable performance, while the performance dropped significantly without residue weight.

### MULoc-target provides comprehensive and accurate targeting peptide classification and detection

We evaluated MULoc-Target's performance in targeting peptide classification and compared it with TargetP 2.0. The evaluation was initially conducted using the TargetP dataset, allowing us to directly cite the reported performance of TargetP from its original publication [9]. During cross-validation, we ensured that samples from the TargetP dataset that overlapped with our training set were excluded from each fold, validating the model using the remaining protein samples. The Matthews correlation coefficient (MCC) and F1 scores for classification predictions are presented in Table 2. The evaluation focused on the four types of targeting peptides within the scope of TargetP 2.0, even though MULoc-Target can predict all eight types of targeting peptides. The TargetP dataset simplifies the classification task by assuming that each protein contains only one of the four targeting peptide types. Additionally, in this dataset, TH refers to the entire N-terminus, disregarding the biological fact that TH always follows CH. As shown in Table 2, MULoc-Target outperformed TargetP in classifying all four targeting peptide types.

**Table 2:** Comparing MULoc-target and TargetP 2 in targeting peptide classification and detection performance using the TargetP dataset.

Targeting peptide	Number of proteins	MULoc-Target			TargetP 2		
		Targeting peptide Classification		Targeting peptide detection	Targeting peptide Classification		Targeting peptide detection
		MCC	F1	Cleavage site accuracy	MCC	F1	Cleavage site accuracy
MT	499	**0.91**	**0.92**	**0.64**	0.86	0.86	0.46
SP	2697	**0.98**	**0.98**	**0.88**	0.97	**0.98**	0.83
CH	227	**0.94**	**0.94**	0.34	0.88	0.88	**0.49**
TH	45	**0.81**	**0.79**	**0.90**	0.75	0.75	0.60

Best results for each task are shown in bold.

We then compared MULoc-Target with TargetP in terms of the ability to detect targeting peptide positions within protein sequences. MULoc-Target demonstrated superior performance in detecting cleavage sites for MT, SP, and TH peptides, but underperformed for CH peptides. This discrepancy is primarily attributed to the biological interplay between CH and TH signals. Thylakoid transit signals always follow chloroplast transit signals within protein sequences. TargetP simplifies such cases by treating them as TH alone since the softmax activation in TargetP model architecture only allows single-label prediction. Meanwhile, MULoc-Target accounts for their natural structural relationship and predicts cleavage sites for both CH and TH signals in these samples. This biologically accurate approach may explain the observed difference in performance for CH signals.

In addition to these four N-terminal targeting peptides, we evaluated MULoc-Target's performance in detecting NES and NLS signals, which can occur anywhere within a protein sequence. For this analysis, we compared MULoc-Target with two specialized tools: INSP (Identification of nucleus signal peptide) [22] for NLS detection and NESmapper [23] for NES detection. The INSP method identifies NLS signals using statistical knowledge and machine learning. INSP treats protein sequences as text, extracting sequence context features through a natural language processing model. These features are integrated with statistical information about the query sequence to construct a multivariate regression model for NLS identification. NESmapper, on the other hand, predicts NES signals by leveraging activity-based profiles of all NES classes, which were generated through comprehensive mutational analyses in mammalian cells. The method combines these profiles with the amino acid properties of NES-flanking regions to optimize predictions using a computational model. Cross-validation was performed on our UniProt-EC7 dataset, ensuring that samples used for training by INSP or NESmapper were excluded from the testing set in each fold.

As shown in Table 3, MULoc-Target outperformed INSP in detecting the positions of NLS, but underperformed in NES detection compared to NESmapper. One contributing factor to NESmapper's superior performance is the use of experiment-derived profiles (mutational analyses), which enhance sensitivity in NES detection. Another potential reason is that, while we excluded exact protein sequences used for NESmapper's training from the testing set, proteins with high sequence identity to the training samples may remain. This overlap could introduce bias favoring the profile-based NESmapper method.

**Table 3:** Comparing performance in NLS and NES detection.

Targeting peptide	Number of proteins	Performance (SOV score)		
		MULoc-Target	INSP	NESmapper
NLS	113	**0.634**	0.556	–
NES	39	0.278	–	**0.452**

Best results for each task are shown in bold.

We further compared MULoc-Target with DeepLoc2 by evaluating targeting peptide classification performance using the sorting signals dataset from the original DeepLoc2 paper [11]. The results for the seven common targeting peptide types between the two methods are summarized in Table 4. The findings indicate that MULoc-Target achieves competitive classification performance compared to DeepLoc2, which is also based on pre-trained language models (PLMs). However, MULoc-Target offers an additional advantage by precisely targeting peptide localization alongside classification, addressing a critical need for comprehensive targeting signal analysis.

**Table 4:** Comparing performance in targeting peptide classification on the sorting signals dataset.

Targeting peptide	Number of proteins	Performance (MCC)		
		MULoc-Target	DeepLoc 2 (ESM1b)	DeepLoc 2 (ProtT5)
PTS	125	0.86 ± 0.05	0.85 ± 0.06	**0.90 ± 0.05**
MT	236	0.90 ± 0.03	**0.93 ± 0.02**	0.93 ± 0.03
SP	768	**0.91 ± 0.03**	0.89 ± 0.03	0.90 ± 0.03
NLS	131	0.59 ± 0.07	0.65 ± 0.06	**0.66 ± 0.01**
NES	83	0.47 ± 0.11	**0.49 ± 0.20**	0.46 ± 0.17
CH	90	**0.96 ± 0.02**	0.85 ± 0.07	0.86 ± 0.09
TH	42	**0.87 ± 0.11**	0.86 ± 0.08	0.80 ± 0.08
Average		**0.7943**	0.7886	0.7871
Weighted average		**0.8547**	0.8469	0.8545

Best results for each task are shown in bold.

Note: The weighted average is based on the sample number of each class in the dataset.

### Precise targeting peptide detection benefits high-resolution mechanistic analysis

**Figure 3:**
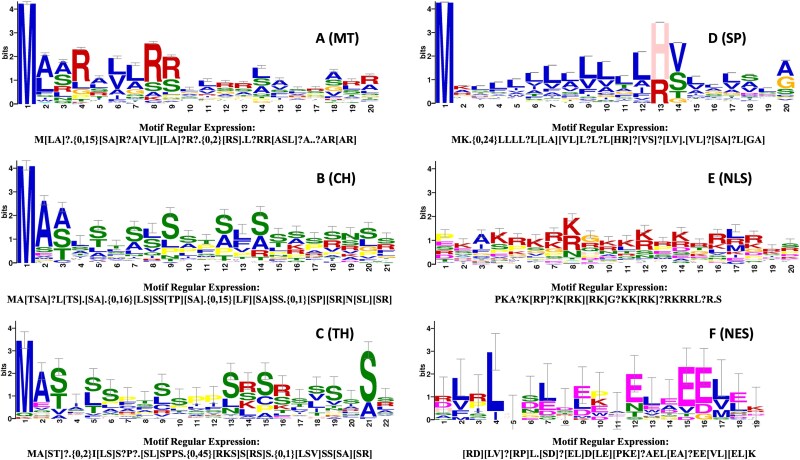
The motif logos and regular expression generated by GLAM2 by aligning the MULoc-target detected targeting peptides in (A) mitochondrial transit peptide; (B) signal peptide; (C) chloroplast transit peptide; (D) nuclear localization signals; (E) thylakoid targeting peptides; (F) nuclear export signals.

To extract targeting peptide patterns and analyze their underlying mechanisms, we previously ranked amino acids based on attention weights and selected protein sequence segments centered on the top-ranked amino acids [12]. With MULoc-Target now capable of detecting precise targeting peptide positions, we could analyze these patterns with a much higher signal-to-noise ratio. For each targeting peptide class, we used MULoc-Target to detect the signals within protein sequences and input these sequence segments into GLAM2 [24], a tool from the MEME Suite 5.1.0, to identify variable-length, gapped motifs. Figure 3 presents the motif logos obtained after aligning the detected targeting peptides for each class, along with the corresponding regular expressions of the motifs. The motifs for ER and PTS are absent since they were too short to be analyzed by GLAM2.

In Fig. 3(A), the mitochondrial transit peptide (MT) motif reveals the frequent presence of arginine (R), with occasional lysine (K), giving the peptide a positive charge critical for interacting with the negatively charged mitochondrial membrane potential. Toward the C-terminal region, clustering of arginine (R) and small residues such as alanine (A), serine (S), or leucine (L) resembles mitochondrial processing peptidase cleavage sites. The characteristic sequences, such as “R near R” (R …R) or "RXXR," are commonly recognized cleavage signals across various organisms [25]. These features—positive charge, amphipathic nature, and identifiable cleavage sites—are hallmarks of mitochondrial targeting peptides [26].

In Fig. 3(B), the chloroplast transit peptide (CH) motif begins with the N-terminal "M-A," reflecting the start codon (methionine) followed by alanine or another small residue, common in many plant precursor proteins [9]. CH peptides are enriched with hydroxylated residues (serine [S], threonine [T]) and small residues (alanine [A]), forming flexible, amphipathic structures that interact with the import machinery [9]. The motif also includes hydrophobic residues (proline [P]) and consecutive serine residues (SS), which may participate in phosphorylation or structural recognition [27]. Near the cleavage site, a combination of serine (S), proline (P), arginine (R), and neutral residues like asparagine (N) or leucine (L) is observed, aligning with the stromal processing peptidase (SPP) cleavage preferences [28].

In Fig. 3(C), thylakoid targeting peptides (TH) motifs share similarities with chloroplast transit peptides, reflecting their two-part targeting system. These peptides often include arginine (R) or lysine (K), positively charged residues critical for specific transport pathways, such as the twin-arginine translocation (Tat) pathway [29]. The tail region often contains small and polar residues like serine (S), alanine (A), or arginine (R), which mark cleavage sites for thylakoid processing peptidase (TPP) [29]. This dual-role system enables proteins to be transferred from the stroma to thylakoid membranes or lumen.

In Fig. 3(D), the core of the signal peptide (SP) motif is a leucine-rich, hydrophobic region (the H-region), flanked by typical features of signal peptides. These features align with the canonical structure of signal peptides [30]: the N-region (at the beginning of the SP), containing positively charged residues like lysine (K) and arginine (R); the H-region (in the middle of the SP), a hydrophobic stretch enriched in leucine (L), isoleucine (I), valine (V), or other nonpolar residues; and the C-region (at the end of the SP), where the cleavage site is recognized by signal peptidases, typically featuring small, neutral residues [31].

In Fig. 3(E), nuclear localization signals (NLS) are defined by clusters of positively charged residues, such as arginine (R) and lysine (K), forming "basic patches" recognized by importin α [32]. Classic monopartite NLS signals, such as KKKRK, consist of a single stretch of basic residues. In some cases, a short tail of additional residues follows the basic cluster, ending with polar or charged residues (e.g. R, S), which may enhance accessibility or enable post-translational modifications [32].

In Fig. 3(F), nuclear export signals (NES) exhibit a hydrophobic anchor motif, most commonly leucine (L), with contributions from isoleucine (I), valine (V), and phenylalanine (F). These hydrophobic residues are interspersed with acidic or polar residues, such as aspartic acid (D) or glutamic acid (E), which may enhance solubility, structural orientation, or regulation through mechanisms like phosphorylation. The pattern often ends with a basic residue, such as lysine (K), which may facilitate electrostatic interactions or serve as a binding site for CRM1, the export receptor [33].

The detailed motifs and structural insights presented in Fig. 3 demonstrate the versatility of MULoc-Target in uncovering biologically meaningful targeting peptide patterns. These findings align with known functional characteristics of the targeting signals and provide a framework for understanding their role in protein sorting mechanisms. To understand how electric charge influences different targeting peptides, we analyzed the motif regular expressions in terms of positively charged residues (K, R, H) and negatively charged residues (D, E). The total electric charges of each motif gave insights into their biological properties, including how they interact with transport machinery, membranes, and receptors. The results are summarized in Table S5. The patterns of amino acid sequences that occur in motifs, as well as the blocks of charged residues, appear overall to be highly constrained [34–36].

### MULoc-target may correct targeting peptide annotation in UniProt

Our UniProt-EC7 dataset includes targeting peptide annotations, many of which lack experimental verification (only entries with the code "ECO269" have experimental evidence). We hypothesize that some discrepancies between MULoc-Target predictions and the database "ground truth" arise from incorrect annotations. This hypothesis is also supported by the results in Table S3, as the performance for some classes improved after removing testing samples without experimental verification (compared to Table 1 for the classes with sufficient verified samples left). The well-studied peroxisomal targeting signal (PTS) mechanism provides an ideal case study to explore this idea. The PEX5 protein serves as a receptor for PTS1, binding to target proteins and facilitating their import into peroxisomes [37]. By using the AlphaFold 3 [38], we can predict the complex structure of PEX5 with its cargo proteins and analyze the binding sites to validate MULoc-Target's predictions. We selected protein samples that met both of the following two criteria: (1) MULoc-Target's predicted PTS position differed from the annotated PTS position in the database. (2) The database annotation lacked experimental verification. Two peroxisomal proteins and their corresponding PEX5 receptors were selected and analyzed: P53164 (NPY1) paired with P35056 (PEX5) in *Saccharomyces cerevisiae*, and Q8LPS1 (LACS6) paired with Q9FMA3 (PEX5) in *Arabidopsis thaliana*. The full list of peroxisomal proteins is shown in Table S6.

**Figure 4:**
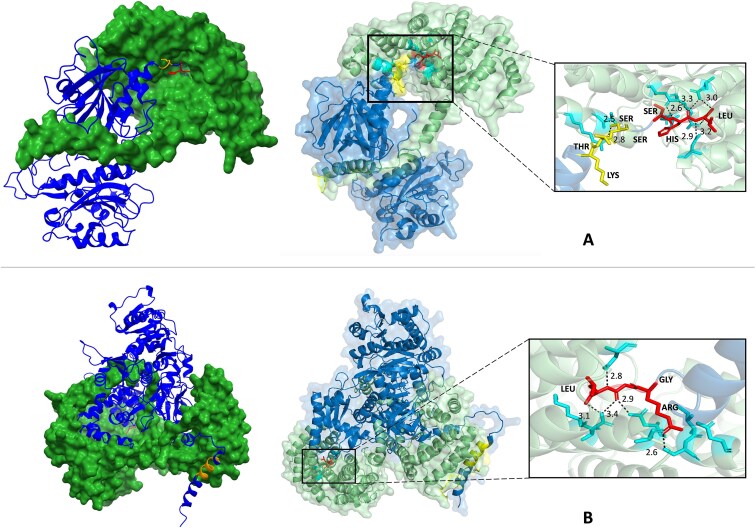
Predicted protein dimer structure by Alphafold 3. (A) Peroxisomal protein NPY1 (P53164, cartoon display) interacting with PEX5 protein (P35056, surface display) in *Saccharomyces cerevisiae*. (B) Peroxisomal protein LACS6 (Q8LPS1, cartoon display) interacting with PEX5 protein (Q9FMA3, surface display) in *Arabidopsis thaliana*. The yellow region is the annotated binding PTS in Uniprot. The red region is the predicted binding PTS by MULoc-target. The dashed lines in the amplified areas are hydrogen bonds between atoms.

For P53164, the annotated PTS is located at amino acids 378–380 (KTS), while MULoc-Target predicted the PTS to be the last three amino acids at the C-terminus (SHL). For Q8LPS1, the annotated PTS spans amino acids 15–23 (RINAIHSHL), whereas MULoc-Target predicted it as the last three amino acids at the C-terminus (RGL). We used the AlphaFold 3 web server to predict the dimer structures of these proteins and their respective PEX5 receptors (see details of AlphaFold3 application in Materials and Methods). The results are shown in Fig. 4.

In Fig. 4(A), MULoc-Target's predicted PTS for P53164 (SHL) is deeply embedded within the PEX5 binding pocket, forming more hydrogen bonds (dashed lines) compared to the annotated PTS. Similarly, in Fig. 4(B), MULoc-Target's predicted PTS for Q8LPS1 (RGL) tightly interacts with the binding pocket formed by PEX5, while the annotated PTS is part of a helix structure distant from the PEX5 protein. We conducted further experiments to confirm our conclusions. Specifically, we identified all peroxisomal proteins with experimentally verified PTS1 signals in *S. cerevisiae* and *A. thaliana*. We then ran AlphaFold3 on these proteins in complex with their corresponding PEX5 proteins. Our analysis revealed that the binding motifs on PEX5 are conserved, as demonstrated in Fig. S1 (which shows PEX5 amino acids binding to verified PTS). Notably, our predictions produced a well-matched pattern, with a strong overlap between PEX5 binding sites and the predicted PTS (Fig. S1), whereas the annotated regions showed poor overlap. Furthermore, when we superimposed the predicted and verified PTS (Fig. S2), we observed numerous conserved hydrogen bonds between them and PEX5, despite variations in their sequences and structures. These findings strongly suggest that MULoc-Target's predictions align more closely with the structural binding mechanism of PTS1. A complete list of the AlphaFold results is summarized in Tables S7 and S8.

These results demonstrate MULoc-Target's ability to identify and correct low-confidence targeting peptide annotations in the database, providing a more accurate representation of targeting peptide positions. This capability highlights MULoc-Target's potential as a tool for refining protein annotations and improving the reliability of publicly available datasets.

### The MULoc-target web server

We have developed a web server for the MULoc-Target method, accessible at https://mu-loc.org/MULoc-Target, and integrated it into our existing MULocDeep protein localization platform as a new feature. This web server offers users a straightforward interface to utilize the MULoc-Target method. Users can upload protein sequences and submit jobs, after which the backend processes the input using MULoc-Target and provides predicted targeting peptide classifications and their precise positions within the query protein sequence, presented as an interactive graphical output. Each user is allocated a personalized workspace within our database, enabling efficient job management. Notably, the MULoc-Target web server is the only platform that delivers both comprehensive targeting peptide classification and precise signal position detection. This new functionality enhances the versatility of our MULocDeep localization platform, offering researchers a more robust and convenient tool for protein localization analysis.

## Discussion and conclusion

In this paper, we introduce MULoc-Target, a deep learning-based method for eukaryotic protein targeting peptide classification and precise position detection (as illustrated in Fig. 1). The model utilizes the protein language model ESM2 as its backbone to encode protein sequences and extract rich contextual information. To fine-tune the model efficiently, we experimented with several parameter-efficient fine-tuning (PEFT) strategies (Fig. 1 and Table 1). Additionally, we designed specialized task heads and an inference procedure to unify the classification and signal detection tasks, eliminating the need for arbitrary threshold determination. This innovative design could be generalized to other sequential motif or signal detection problems, broadening its applicability.

We also curated and provided a benchmark dataset, UniProt-EC7, which includes eight types of eukaryotic protein targeting peptides with manually curated annotations. The dataset is divided into five folds with sequence identity control to ensure robust evaluation. This benchmark dataset is intended to serve as a valuable resource for the research community, facilitating the development and comparison of future methods.

We evaluated our method using the UniProt-EC7 benchmark dataset, as well as several external datasets from the literature. All the compared methods have the same taxonomy scope (eukaryotes). The results demonstrate that MULoc-Target achieves superior or competitive performance in both targeting peptide classification and detection tasks compared to state-of-the-art methods (Tables 2–4). The only exception is in NES detection, where a specialized hybrid method combining experimental profiles and computational techniques achieved a higher SOV score. This underscores the potential of profile-based approaches for specific niche tasks.

MULoc-Target’s ability to detect targeting peptides with high accuracy allows for the extraction and alignment of motifs, providing insights into their properties and underlying mechanisms. Such knowledge would guide the generation of novel protein sequences that promote selective subcellular distribution, and the analysis of mutation effects on protein localization [39]. The enriched motif logos and corresponding regular expressions generated by MULoc-Target align well with existing knowledge from targeting peptide studies (Fig. 3). Moreover, cases where MULoc-Target's predictions differed from database annotations were often supported by tools such as AlphaFold 3 (Fig. 4). These results indicate that MULoc-Target can potentially correct low-quality or inaccurate targeting peptide annotations in existing databases.

While MULoc-Target has shown promising results, there is room for further improvement, especially for classes with small sample sizes. Future work could explore other protein language models, such as ProtT5 [40], which demonstrated superior performance in DeepLoc2 compared to ESM1b. The latest third-generation ESM models [36] could also enhance representation capabilities. Another promising direction is incorporating additional data modalities, such as protein structure and protein–protein interaction information, into the framework. Such information could improve targeting peptide detection, as these features are integral to protein sorting mechanisms. Our in-house structure-ware protein language model (SPLM) [41] can be utilized for this purpose, as it integrates protein structure information into protein sequence embeddings.

In summary, MULoc-Target represents a significant step forward in understanding protein targeting mechanisms by integrating advanced deep learning techniques with carefully curated datasets. By making the datasets, software code, and the MULoc-Target website publicly accessible, this study aims to foster further research and innovation in protein sorting and targeting peptide detection.

**Key Points**

- We proposed a protein language model based deep learning model, MULoc-Target, to classify protein targeting peptides and detect their precise position in protein sequences. It achieves state-of-the-art performance for both tasks.
- MULoc-Target covers eight main protein targeting peptide classes, which makes it the most systematic and comprehensive targeting peptide prediction and detection tool to date. The model has been integrated into the MULocDeep localization web server for easy access.
- MULoc-Target enables the extraction of the enriched targeting peptide patterns, offering valuable insights into their properties and the underlying targeting mechanisms.

## Supplementary Material

supplementary_submit_bbaf436

## Data Availability

The codes and datasets are available online at https://github.com/yuexujiang/MULoc-Target.
