# Supplementary material for: MULoc-target: Targeting peptide classification and detection using a protein language model
Source: Brief Bioinform. 2025 Aug 27;26(4):bbaf436. doi: 10.1093/bib/bbaf436 (PMC12454934; doi:10.1093/bib/bbaf436)
Supplement: supplementary_submit_bbaf436 [file supplementary_submit_bbaf436.docx]

| **Table S1**. Number of samples in each targeting peptide class | |
| --- | --- |
| Targeting peptide classes | Sample counts |
| Signal peptide (SP) | 3226 |
| Nuclear localization signals (NLS) | 826 |
| Mitochondrial transit peptides (MT) | 793 |
| Chloroplast transit peptides (CH) | 351 |
| Nuclear export signals (NES) | 159 |
| Endoplasmic reticulum retention signal (ER) | 146 |
| Peroxisomal targeting signals (PTS) | 62 |
| Thylakoidal transit peptides (TH) | 46 |

| **Table S2**. Number of samples by detailed evidence code | | |
| --- | --- | --- |
| Targeting peptide classes | Evidence codes of targeting peptide annotation | Sample counts |
| ER | ECO:0000305\|yy | 1 |
| ER | ECO:0000269\|yy | 6 |
| ER | ECO:0000255\|yy | 131 |
| ER | ECO:0000250\|yy | 8 |
| Mitochondrion | ECO:0007744\|yy | 21 |
| Mitochondrion | ECO:0000305\|yy | 14 |
| Mitochondrion | ECO:0000303\|yy | 1 |
| Mitochondrion | ECO:0000269\|yy | 522 |
| Mitochondrion | ECO:0000255\|yy | 74 |
| Mitochondrion | ECO:0000255, ECO:0007744\|yy | 8 |
| Mitochondrion | ECO:0000255, ECO:0000305\|yy | 2 |
| Mitochondrion | ECO:0000255, ECO:0000269\|yy | 2 |
| Mitochondrion | ECO:0000250\|yy | 149 |
| Nucleus | ECO:0000305\|yy | 32 |
| Nucleus | ECO:0000303\|yy | 7 |
| Nucleus | ECO:0000269\|yy | 209 |
| Nucleus | ECO:0000255\|yy | 367 |
| Nucleus | ECO:0000255, ECO:0000305\|yy | 1 |
| Nucleus | ECO:0000255, ECO:0000303\|yy | 1 |
| Nucleus | ECO:0000255, ECO:0000269\|yy | 1 |
| Nucleus | ECO:0000250\|yy | 208 |
| Nucleus_export | ECO:0000305\|yy | 8 |
| Nucleus_export | ECO:0000269\|yy | 58 |
| Nucleus_export | ECO:0000255\|yy | 8 |
| Nucleus_export | ECO:0000250\|yy | 85 |
| Peroxisome | ECO:0007744\|yy | 1 |
| Peroxisome | ECO:0000305\|yy | 9 |
| Peroxisome | ECO:0000269\|yy | 24 |
| Peroxisome | ECO:0000255\|yy | 4 |
| Peroxisome | ECO:0000255, ECO:0000305\|yy | 1 |
| Peroxisome | ECO:0000255, ECO:0000303\|yy | 1 |
| Peroxisome | ECO:0000250\|yy | 22 |
| SIGNAL | ECO:0007744\|yy | 5 |
| SIGNAL | ECO:0000305\|yy | 70 |
| SIGNAL | ECO:0000303\|yy | 4 |
| SIGNAL | ECO:0000269\|yy | 2819 |
| SIGNAL | ECO:0000255\|yy | 19 |
| SIGNAL | ECO:0000255, ECO:0007744\|yy | 8 |
| SIGNAL | ECO:0000255, ECO:0000312\|yy | 16 |
| SIGNAL | ECO:0000255, ECO:0000305\|yy | 8 |
| SIGNAL | ECO:0000255, ECO:0000303\|yy | 10 |
| SIGNAL | ECO:0000255, ECO:0000269\|yy | 20 |
| SIGNAL | ECO:0000250\|yy | 247 |
| Thylakoid | ECO:0000305\|yy | 2 |
| Thylakoid | ECO:0000269\|yy | 42 |
| Thylakoid | ECO:0000250\|yy | 2 |
| chloroplast | ECO:0007744\|yy | 72 |
| chloroplast | ECO:0000305\|yy | 8 |
| chloroplast | ECO:0000305, ECO:0007744\|yy | 4 |
| chloroplast | ECO:0000303\|yy | 2 |
| chloroplast | ECO:0000269\|yy | 220 |
| chloroplast | ECO:0000255\|yy | 14 |
| chloroplast | ECO:0000255, ECO:0007744\|yy | 20 |
| chloroplast | ECO:0000250\|yy | 11 |
| The “yy” indicates arbitrary reference source for the annotation | | |

| **Table S3**. Cross-validation performance of MULoc-Target on the ECO:0000269 subset of the UniProt-EC7 dataset | | | | |
| --- | --- | --- | --- | --- |
| Targeting peptide | Sample counts | Classification | | Targeting peptide detection |
|  |  | MCC | F1 | Cleavage site accuracy |
| ER | 8 | 0.544 | 0.477 | 0.978 |
| PTS | 24 | 0.461 | 0.457 | 0.800 |
| MT | 529 | 0.941 | 0.948 | 0.620 |
| SP | 2840 | 0.955 | 0.987 | 0.878 |
| CH | 177 | 0.963 | 0.965 | 0.344 |
| TH | 42 | 0.807 | 0.794 | 0.950 |
|  |  | MCC | F1 | SOV score |
| NLS | 185 | 0.667 | 0.660 | 0.661 |
| NES | 47 | 0.429 | 0.379 | 0.314 |
| Average |  | 0.721 | 0.708 | 0.693 |
| Weighted average |  | 0.928 | 0.951 | 0.801 |
| Note: The weighted average is based on the sample number of each class in the dataset. | | | | |

| **Table S4**. Ablation test of different model configurations | | | | | | | | | | | | |
| --- | --- | --- | --- | --- | --- | --- | --- | --- | --- | --- | --- | --- |
| Targeting peptide | Model configurations | | | | | | | | | | | |
|  | Using DA+CW+RW | | | Without CW | | | Without RW | | | Without DA | | |
|  | MCC | F1 | Cleavage site accuracy | MCC | F1 | Cleavage site accuracy | MCC | F1 | Cleavage site accuracy | MCC | F1 | Cleavage site accuracy |
| ER | 0.72 | 0.7 | 1 | 0.71 | 0.70 | 1 | **0.75** | **0.73** | 0 | 0.72 | 0.71 | 1 |
| Peroxisome | **0.78** | **0.78** | 1 | 0.77 | 0.76 | 1 | 0.26 | 0.22 | 0 | 0.71 | 0.67 | 1 |
| Mitochondrion | 0.92 | 0.93 | **0.60** | **0.95** | **0.95** | 0.51 | 0.07 | 0.01 | 0 | 0.93 | 0.94 | 0.56 |
| Signal peptide | 0.94 | 0.98 | 0.87 | **0.95** | 0.98 | 0.86 | 0.86 | 0.94 | **0.88** | 0.94 | 0.98 | 0.86 |
| Chloroplast | 0.98 | 0.98 | 0.32 | **0.99** | **0.99** | 0.37 | 0.91 | 0.92 | 0 | 0.97 | 0.97 | **0.42** |
| Thylakoid | 0.80 | 0.78 | 1 | 0.80 | 0.78 | 1 | 0.77 | 0.75 | 0 | 0.8 | 0.78 | 1 |
|  | MCC | F1 | SOV score | MCC | F1 | SOV score | MCC | F1 | SOV score | MCC | F1 | SOV score |
| NLS | 0.74 | 0.77 | **0.66** | **0.81** | **0.84** | 0.61 | 0.63 | 0.63 | 0.16 | 0.76 | 0.79 | 0.63 |
| NES | 0.46 | 0.45 | **0.27** | 0.44 | 0.44 | 0.19 | 0.47 | 0.44 | 0.06 | **0.51** | **0.52** | 0.18 |
| Average | 0.79 | 0.80 | **0.72** | **0.80** | **0.81** | 0.69 | 0.59 | 0.58 | 0.14 | 0.79 | 0.80 | 0.71 |
| Weighted average | 0.89 | 0.92 | **0.76** | **0.91** | **0.93** | 0.73 | 0.70 | 0.73 | 0.53 | 0.89 | 0.92 | 0.74 |
| DA: Data augmentation  CW: Class weight  RW: residue weight  Note: The evaluation is based on one fold instead of cross-validation. The weighted average is based on the sample number of each class in the dataset. | | | | | | | | | | | | |

| **Table S5**. Charge characterization of each targeting peptide motif | | | | |
| --- | --- | --- | --- | --- |
| Peptide | Motif Pattern | Residues | Net charge | Biological Insight |
| Mitochondrial (MT) | M[LA]?.{0,15}[SA]R?A[VL][LA]?R?.{0,2}[RS].L?RR[ASL]?A..?AR[AR] | Arginine (R) enriched | Highly positive  2.87 $\pm$ 1.02 | Strong affinity to the negatively charged mitochondrial membrane, facilitating import |
| Chloroplast (CH) | MA[TSA]?L[TS].[SA].{0,16}[LS]SS[TP][SA].{0,15}[LF][SA]SS.{0,1}[SP][SR]N[SL][SR] | Few charged residues | Mostly neutral  1.79 $\pm$ 1.36 | Weak charge makes it distinct from MTP, interacts with TOC/TIC complexes for chloroplast import |
| Thylakoid (TH) | MA[ST]?.{0,2}I[LS]S?P?.[SL]SPPS.{0,45}[RKS]S[RS]S.{0,1}[LSV]SS[SA][SR] | Moderate R, K content | Weakly positive  0.95 $\pm$ 0.96 | Works alongside CH, often involving Tat pathway (twin-arginine signal) |
| Signal Peptide (SP) | MK.{0,24}LLLL?L[LA][VL]L?L?L[HR]?[VS]?[LV].[VL]?[SA]?L[GA] | Mildly positive (K, H, R) | Slightly positive to neutral  0.65 $\pm$ 0.79 | Weak positive charge aids signal recognition particle (SRP) binding and ER import |
| Nuclear Localization (NLS) | PKA?K[RP]?K[RK][RK]G?KK[RK]?RKRRL?R.S | Enriched in lysine (K) and arginine (R) | Strongly positive  3.89 $\pm$ 1.32 | High charge aids importin recognition for nuclear transport |
| Nuclear Export (NES) | [RD][LV]?[RP]L.[SD]?[EL]D[LE][PKE]?AEL[EA]?EE[VL][EL]K | Rich in glutamate (E) and aspartate (D) | Negative charge  -0.34 $\pm$ 1.52 | Facilitates CRM1-mediated nuclear export, interacting with exportins |
| Note: the net charge calculates the charge sum on each motif, then calculates the mean $\pm$ standard deviation for the motifs in each class. | | | | |

| **Table S6.** List of peroxisomal proteins from the UniProt-EC7 dataset with their annotated and predicted peroxisomal targeting signal (PTS) | | | | | |
| --- | --- | --- | --- | --- | --- |
| Entry | Annotated PTS indices in database | Evidence | Targeting peptide prediction match | Cleavage site prediction match | Predicted PTS |
| F7VNF8 | 907-909 | ECO:0000255\|HAMAP-Rule:MF_03121 | TRUE | TRUE | SRL |
| O09174 | 379-381 | ECO:0000250\|UniProtKB:Q9UHK6 | TRUE | TRUE | ANL |
| O15254 | 698-700 | ECO:0000250\|UniProtKB:Q63448 | TRUE | TRUE | SKL |
| P08679 | 458-460 | ECO:0000269\|PubMed:2181273 | TRUE | TRUE | SKL |
| P30044 | 212-214 | ECO:0000305\|PubMed:10514471 | TRUE | TRUE | SQL |
| P30952 | 552-554 | ECO:0000269\|PubMed:11846793 | TRUE | TRUE | SKL |
| P32020 | 545-547 | ECO:0000255, ECO:0000303\|PubMed:26901662 | TRUE | TRUE | AKL |
| P38137 | 541-543 | ECO:0000269\|PubMed:26359497 | TRUE | TRUE | SKL |
| P38225 | 667-669 | ECO:0000305\|PubMed:22493507 | TRUE | TRUE | IKL |
| P38998 | 371-373 | ECO:0000255, ECO:0000305\|PubMed:10077615 | TRUE | TRUE | SRL |
| P39518 | 742-744 | ECO:0000305\|PubMed:8670886 | TRUE | TRUE | EKL |
| P53164 | 378-380 | ECO:0000250\|UniProtKB:Q9BQG2 | TRUE | FALSE | SHL |
| P99029 | 208-210 | ECO:0000250\|UniProtKB:P30044 | TRUE | TRUE | SQL |
| Q08558 | 269-271 | ECO:0000269\|PubMed:10639339, ECO:0000269\|PubMed:11302517 | TRUE | TRUE | HKL |
| Q12524 | 1-7 | ECO:0000269\|PubMed:10922370 | TRUE | FALSE | SLW |
| Q3MIB4 | 850-852 | ECO:0000255\|HAMAP-Rule:MF_03121 | TRUE | TRUE | SKL |
| Q4WF54 | 268-270 | ECO:0000269\|PubMed:23617799 | TRUE | TRUE | SKL |
| Q4WF55 | 460-462 | ECO:0000269\|PubMed:23617799 | TRUE | TRUE | AKL |
| Q63448 | 698-700 | ECO:0000269\|PubMed:8706733 | TRUE | TRUE | SQL |
| Q86WA8 | 850-852 | ECO:0000255\|HAMAP-Rule:MF_03121 | TRUE | TRUE | SKL |
| Q8CHM7 | 579-581 | ECO:0000250\|UniProtKB:Q9UJ83 | TRUE | TRUE | SNI |
| Q8GW43 | 474-476 | ECO:0000269\|PubMed:21730067 | TRUE | TRUE | PKL |
| Q8LPS1 | 15-23 | ECO:0000305\|PubMed:12481085 | TRUE | FALSE | RGL |
| Q9BQG2 | 460-462 | ECO:0000305\|PubMed:12790796 | TRUE | TRUE | PNL |
| Q9DBN5 | 850-852 | ECO:0000255\|HAMAP-Rule:MF_03121 | TRUE | TRUE | SKL |
| Q9DCN1 | 460-462 | ECO:0000250\|UniProtKB:Q9BQG2 | TRUE | TRUE | PNL |
| Q9EPL9 | 698-700 | ECO:0000250\|UniProtKB:Q63448 | TRUE | TRUE | SQL |
| Q9FWA3 | 484-486 | ECO:0000305\|PubMed:26941195 | TRUE | TRUE | SKI |
| Q9QXE0 | 579-581 | ECO:0000250\|UniProtKB:Q9UJ83 | TRUE | TRUE | SNM |
| Q9SIP1 | 261-263 | ECO:0000305\|PubMed:24130194 | TRUE | TRUE | SSL |
| Q9UHK6 | 380-382 | ECO:0000269\|PubMed:11060344 | TRUE | TRUE | ASL |
| Q9UJ83 | 576-578 | ECO:0000269\|PubMed:10468558 | TRUE | TRUE | SNM |
| Note: the proteins are highlighted if 1) the predicted PTS does not match the annotated PTS in the UniProt database. And 2) The PTS annotation is not experimentally verified. The entries in red correspond to the two proteins in Fig. 4. | | | | | |

| **Table S7.** AlphaFold3 predicted top 5 structures for each dimer complex (peroxisomal protein-PEX5) in *Saccharomyces cerevisiae* | | | | | |
| --- | --- | --- | --- | --- | --- |
| PTS | Peroxisomal protein ID | AlphaFold predicted structure index | PTS AAs | Binding AA indices on PEX5 | PTS binding AA indices on peroxisomal protein |
| PTS with experimental verification | P08679 | 0 | SKL | 361, 363, 389, 392, 393, 394, 465, 468, 480, 492, 495, 496, 499, 500, 502, 503, 514, 526, 529, 530, 533, 534, 576 | 458, 459, 460 |
|  |  | 1 | SKL | 361, 363, 389, 392, 393, 394, 465, 468, 480, 492, 495, 496, 499, 500, 502, 503, 514, 526, 530, 533, 534, 576 | 458, 459, 460 |
|  |  | 2 | SKL | 361, 363, 389, 392, 393, 394, 465, 468, 480, 492, 495, 496, 499, 500, 502, 503, 514, 526, 530, 533, 534 | 458, 459, 460 |
|  |  | 3 | SKL | 361, 363, 389, 392, 393, 394, 465, 468, 480, 492, 495, 496, 499, 500, 502, 503, 514, 526, 530, 533, 534 | 458, 459, 460 |
|  |  | 4 | SKL | 361, 363, 389, 392, 393, 394, 465, 468, 480, 492, 495, 496, 499, 500, 502, 503, 514, 526, 530, 533, 534 | 458, 459, 460 |
|  | P30952 | 0 | SKL |  |  |
|  |  | 1 | SKL |  |  |
|  |  | 2 | SKL |  |  |
|  |  | 3 | SKL |  |  |
|  |  | 4 | SKL |  |  |
|  | P38137 | 0 | SKL | 361, 363, 389, 392, 393, 394, 465, 468, 480, 492, 495, 496, 499, 500, 502, 503, 514, 526, 529, 530, 533, 534, 576 | 541, 542, 543 |
|  |  | 1 | SKL | 361, 363, 389, 392, 393, 394, 465, 468, 480, 492, 495, 496, 499, 500, 502, 503, 514, 526, 529, 530, 533, 534, 576 | 541, 542, 543 |
|  |  | 2 | SKL | 361, 363, 389, 392, 393, 394, 465, 468, 480, 492, 495, 496, 499, 500, 502, 503, 514, 526, 529, 530, 533, 534, 576 | 541, 542, 543 |
|  |  | 3 | SKL | 361, 363, 389, 392, 393, 394, 465, 468, 480, 492, 495, 496, 499, 500, 502, 503, 514, 526, 530, 533, 534 | 541, 542, 543 |
|  |  | 4 | SKL | 361, 363, 389, 392, 393, 394, 465, 468, 480, 492, 495, 496, 499, 500, 502, 503, 514, 526, 530, 533, 534 | 541, 542, 543 |
|  | P39994 | 0 | PRL | 361, 363, 389, 392, 393, 394, 465, 468, 480, 492, 495, 496, 499, 500, 502, 503, 514, 526, 530, 533, 576 | 558, 559, 560 |
|  |  | 1 | PRL | 361, 363, 389, 392, 393, 394, 465, 468, 480, 492, 495, 496, 499, 500, 502, 503, 514, 526, 530, 533, 573 | 558, 559, 560 |
|  |  | 2 | PRL | 361, 363, 389, 392, 393, 394, 465, 468, 480, 492, 495, 496, 499, 500, 502, 503, 514, 526, 530, 533, 573 | 558, 559, 560 |
|  |  | 3 | PRL | 285 | 559 |
|  |  | 4 | PRL |  |  |
|  | P47148 | 0 | VKL | 280, 281, 282, 283 | 282 |
|  |  | 1 | VKL | 279, 282, 283, 286 | 282 |
|  |  | 2 | VKL | 251, 254 | 282 |
|  |  | 3 | VKL | 286 | 282 |
|  |  | 4 | VKL | 286 | 282 |
|  | Q08558 | 0 | HKL | 361, 363, 389, 392, 393, 394, 465, 468, 480, 492, 495, 496, 499, 500, 502, 503, 514, 526, 530, 533, 534, 537 | 269, 270, 271 |
|  |  | 1 | HKL | 361, 363, 389, 392, 393, 394, 465, 468, 480, 492, 495, 496, 499, 500, 502, 503, 514, 526, 530, 533, 534, 537 | 269, 270, 271 |
|  |  | 2 | HKL | 361, 363, 389, 392, 393, 394, 465, 468, 480, 492, 495, 496, 499, 500, 502, 503, 514, 526, 530, 533, 534, 537 | 269, 270, 271 |
|  |  | 3 | HKL | 361, 363, 389, 392, 393, 394, 465, 468, 480, 492, 495, 496, 499, 500, 502, 503, 514, 526, 530, 533, 534, 537 | 269, 270, 271 |
|  |  | 4 | HKL | 361, 363, 389, 392, 393, 394, 465, 468, 480, 492, 495, 496, 499, 500, 502, 503, 514, 526, 530, 533, 534, 537 | 269, 270, 271 |
| MULoc-Target predicted PTS | P53164 | 0 | SHL | 361, 363, 389, 392, 393, 465, 468, 480, 492, 495, 496, 499, 500, 502, 503, 514, 526, 529, 530, 533, 534, 576 | 382, 383, 384 |
|  |  | 1 | SHL | 361, 389, 392, 393, 465, 468, 480, 492, 495, 496, 499, 500, 502, 503, 514, 526, 529, 530, 533, 534, 576 | 382, 383, 384 |
|  |  | 2 | SHL | 361, 389, 392, 393, 465, 468, 480, 492, 495, 496, 499, 500, 502, 503, 514, 526, 529, 530, 533, 534, 576 | 382, 383, 384 |
|  |  | 3 | SHL | 361, 363, 389, 392, 393, 465, 468, 480, 492, 495, 496, 499, 500, 502, 503, 514, 526, 529, 530, 533, 534, 576 | 382, 383, 384 |
|  |  | 4 | SHL | 361, 389, 392, 393, 465, 468, 480, 492, 495, 496, 499, 500, 502, 503, 514, 526, 529, 530, 533, 534, 576 | 382, 383, 384 |
| PTS without experimental verification | P53164 | 0 | KTS | 529, 533, 536, 537, 580, 583 | 378, 379, 380 |
|  |  | 1 | KTS | 529, 533, 536, 537, 580, 583 | 378, 379, 380 |
|  |  | 2 | KTS | 285, 529, 533, 536, 537, 580, 583 | 378, 379, 380 |
|  |  | 3 | KTS | 529, 533, 536, 537, 580, 583 | 378, 379, 380 |
|  |  | 4 | KTS | 529, 533, 536, 537, 580, 583 | 378, 379, 380 |
| The blank rows indicate that using atomic distance cutoff=5, no binding amino acids between PEX5 and peroxisome protein were found. | | | | | |

| **Table S8**. AlphaFold3 predicted top 5 structures for each dimer complex (peroxisomal protein-PEX5) in *Arabidopsis thaliana* | | | | | |
| --- | --- | --- | --- | --- | --- |
|  | | | | | |
| PTS | Peroxisomal protein ID | AlphaFold predicted structure index | PTS AAs | Binding AA indexes on PEX5 | PTS binding AA indexes on peroxisomal protein |
| PTS with experimental verification | Q8GW43 | 0 | PKL | 505, 507, 533, 536, 537, 538, 598, 601, 613, 625, 628, 629, 632, 633, 635, 636, 647, 657, 659, 663, 666, 670, 693 | 474, 475, 476 |
|  |  | 1 | PKL | 505, 507, 533, 536, 537, 538, 598, 601, 613, 625, 628, 629, 632, 633, 635, 636, 647, 657, 659, 663, 666, 670, 693 | 474, 475, 476 |
|  |  | 2 | PKL | 505, 506, 507, 533, 536, 537, 598, 601, 613, 625, 628, 629, 632, 633, 635, 636, 647, 657, 659, 663, 666, 670, 693 | 474, 475, 476 |
|  |  | 3 | PKL | 505, 506, 507, 533, 536, 537, 538, 598, 601, 613, 625, 628, 629, 632, 633, 635, 636, 647, 657, 659, 663, 666, 670, 693, 697 | 474, 475, 476 |
|  |  | 4 | PKL | 505, 506, 507, 533, 536, 537, 538, 598, 601, 613, 625, 628, 629, 632, 633, 635, 636, 647, 657, 659, 663, 666, 670, 693 | 474, 475, 476 |
|  | Q84WW2 | 0 | SKL |  |  |
|  |  | 1 | SKL |  |  |
|  |  | 2 | SKL |  |  |
|  |  | 3 | SKL |  |  |
|  |  | 4 | SKL |  |  |
|  | Q8LF36 | 0 | SSL | 505, 507, 533, 536, 537, 598, 601, 613, 625, 628, 629, 632, 633, 635, 636, 647, 657, 659, 663, 666, 667, 670 | 490, 491, 492 |
|  |  | 1 | SSL | 505, 507, 533, 536, 537, 598, 601, 613, 625, 628, 629, 632, 633, 635, 636, 647, 657, 659, 663, 666, 667, 670 | 490, 491, 492 |
|  |  | 2 | SSL | 505, 507, 533, 536, 537, 598, 601, 613, 625, 628, 629, 632, 633, 635, 636, 647, 657, 659, 663, 666, 667, 670 | 490, 491, 492 |
|  |  | 3 | SSL | 505, 507, 533, 536, 537, 598, 601, 613, 625, 628, 629, 632, 633, 635, 636, 647, 657, 659, 663, 666, 667, 670 | 490, 491, 492 |
|  |  | 4 | SSL | 505, 507, 533, 536, 537, 598, 601, 613, 625, 628, 629, 632, 633, 635, 636, 647, 657, 659, 663, 666, 667, 670 | 490, 491, 492 |
| MULoc-Target predicted PTS | Q8LPS1 | 0 | RGL | 375, 378 | 701 |
|  |  | 1 | RGL | 505, 507, 533, 536, 537, 598, 601, 613, 625, 628, 629, 631, 632, 633, 635, 636, 640, 643, 647, 657, 659, 663, 666, 667, 670 | 699, 700, 701 |
|  |  | 2 | RGL |  |  |
|  |  | 3 | RGL | 1, 2, 3, 4, 5, 6, 505, 537, 601, 625, 628, 629, 632, 633, 635, 636, 640, 647, 657, 659, 663, 666, 667, 670, 697 | 699, 700, 701 |
|  |  | 4 | RGL |  |  |
| PTS without experimental verification | Q8LPS1 | 0 | RINAIHSHL | 669, 674, 700, 703, 704, 708 | 15, 16, 19 |
|  |  | 1 | RINAIHSHL | 153, 187, 188, 190, 191, 194, 195, 197, 198, 199, 200, 201, 202, 203, 204 | 15, 16, 18, 19, 22, 23 |
|  |  | 2 | RINAIHSHL |  |  |
|  |  | 3 | RINAIHSHL | 153, 155, 201 | 15, 16, 19 |
|  |  | 4 | RINAIHSHL |  |  |
| The blank rows indicate that using atomic distance cutoff=5, no binding amino acids between PEX5 and peroxisome protein were found. | | | | | |


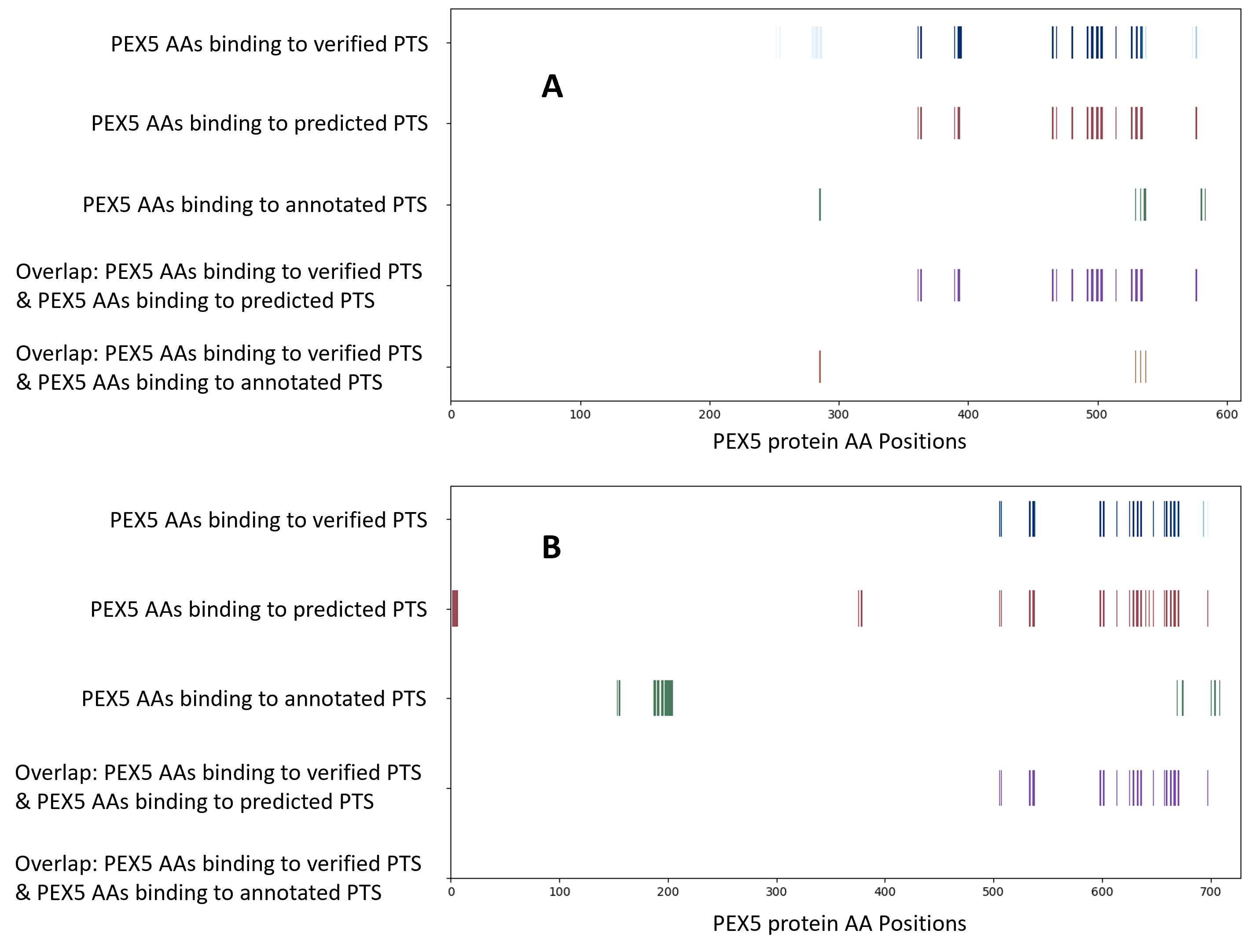


**Figure S1**. The binding amino acid patterns on PEX5 proteins in (A) *Saccharomyces cerevisiae*, and (B) *Arabidopsis thaliana*. The color density of the amino acids binding to the verified PTS (peroxisomal targeting signal) is based on the amino acid frequency in the top 5 structures predicted by AlphaFold as shown in Tables S7 and S8.


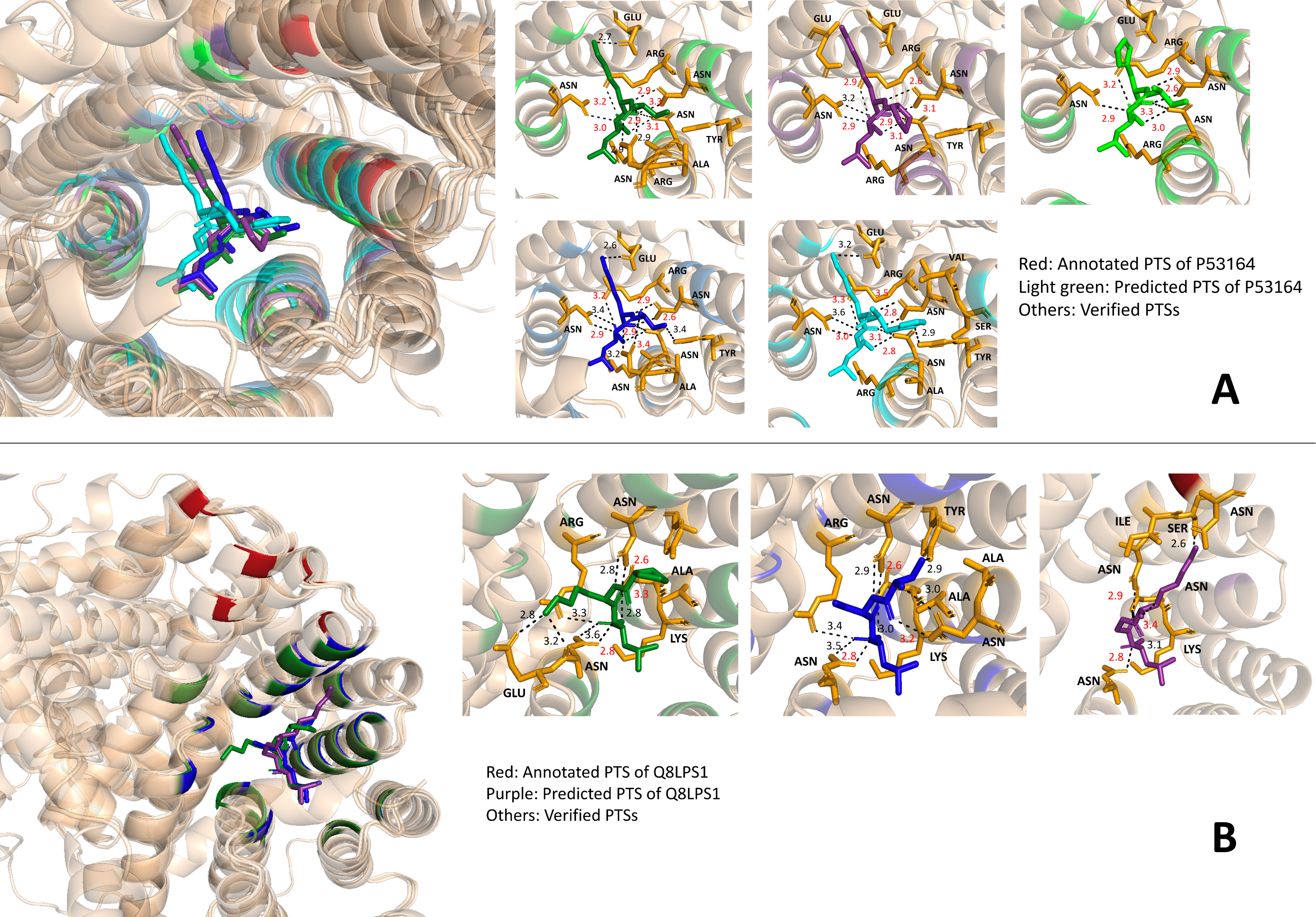


**Figure S2**. The super-imposed and individual PTS (peroxisomal targeting signals) in (A) *Saccharomyces cerevisiae*, and (B) *Arabidopsis thaliana*. The predicted and verified PTS have similar structural pattern and conserved hydrogen binds (red dashed lines), The annotated PTSs don’t follow this verified pattern, which possibly indicates they are misannotated.
